# Biphasic Medium Using Nicotinamide for Detection of Pyrazinamide Resistance in *Mycobacterium tuberculosis*

**DOI:** 10.3390/antibiotics13060563

**Published:** 2024-06-16

**Authors:** Waraporn Thuansuwan, Charoen Chuchottaworn, Chie Nakajima, Yasuhiko Suzuki, Nuntaree Chaichanawongsaroj

**Affiliations:** 1 Program of Molecular Sciences in Medical Microbiology and Immunology, Department of Transfusion Medicine and Clinical Microbiology, Faculty of Allied Health Sciences, Chulalongkorn University, Bangkok 10330, Thailand; w.thuansuwan@gmail.com; 2 Central Chest Institute of Thailand, Bangkasor, Muang, Nonthaburi 11000, Thailand; charojnj@hotmail.com; 3 International Institute for Zoonosis Control, Hokkaido University, Sapporo 001-0020, Japan; cnakajim@czc.hokudai.ac.jp (C.N.); suzuki@czc.hokudai.ac.jp (Y.S.); 4 Research Unit of Innovative Diagnosis of Antimicrobial Resistance, Department of Transfusion Medicine and Clinical Microbiology, Faculty of Allied Health Sciences, Chulalongkorn University, Pathumwan, Bangkok 10330, Thailand

**Keywords:** biphasic media, pyrazinamide, nicotinamide, *pncA*, *Mycobacterium tuberculosis*

## Abstract

Reliable drug susceptibility testing of pyrazinamide (PZA) is technically difficult, since PZA activity is pH sensitive. The aim of this study was to evaluate a biphasic medium assay (BMA) for the reliable detection of PZA resistance in *Mycobacterium tuberculosis* (MTB) using nicotinamide (NIC) as a surrogate for PZA and identifying the appropriate cut-off value for the assay. The PZA susceptibility of 122 multidrug-resistant tuberculosis (MDR-TB) isolates and 39 drug-susceptible tuberculosis (DS-TB) isolates was examined using the BMA with NIC at four different concentrations (250, 500, 1000, and 2000 mg/L) and comparing the results with results from the BACTEC MGIT 960 reference method. Out of 122 MDR-TB isolates, 40 were identified as resistant by the BACTEC MGIT 960 system, of which 92.5% contained mutations within their *pncA* gene plus promoter region. A minimum inhibitory concentration of NIC ≥ 1000 mg/L was used as the cut-off concentration to define resistance in correlation with the MGIT 960 outcomes. NIC-BMA had a sensitivity of 90.91%, a specificity of 100%, and an accuracy of 97.52% compared with the MGIT 960 method. NIC-BMA is a promising assay to screen PZA resistance in microbiological laboratories without automation or advanced molecular instruments.

## 1. Introduction

Tuberculosis, caused by *Mycobacterium tuberculosis,* remains a problem in terms of cure management and control due to incorrect drug prescription practices, patient nonadherence to multiple antibiotic regimens, long therapy periods, and the emergence of multidrug-resistant tuberculosis (MDR-TB) and extensively drug-resistant tuberculosis (XDR-TB) strains [1,2]. 

Pyrazinamide (PZA) is a frontline antituberculosis drug recommended by the World Health Organization for treating both susceptible tuberculosis and MDR-TB. Because of its activity against latent TB that is not killed by other tuberculosis drugs, it has become essential in all new tuberculosis regimens for shortening the treatment from 9 months to 6 months and decreasing the relapse rate [3,4]. However, a high prevalence of PZA resistance has been reported; 16.2% and 60.5% of global tuberculosis and MDR-TB cases were PZA-resistant, respectively [5]. A potential reason for these high PZA-resistance rates is the widespread use of PZA without drug susceptibility testing for the guidance of appropriate regimens [4,6].

PZA is a prodrug that is converted to the active form of pyrazinoic acid (POA) by pyrazinamidase (PZase) encoded by the *pncA* gene [3]. POA inhibits protein and fatty acid synthesis, which are critical for bacterial survival. Most PZA-resistant strains contain *pncA* mutations, causing the reduction or loss in enzyme activity [3,5]. About 72% to 99% of PZA resistance is associated with mutations in *pncA* [7]. However, the genotypic detection of *pncA* mutations is not feasible because of their diversity and distribution throughout the entire open reading frame and its promoter region [5,8].

Phenotypic PZA drug susceptibility testing (DST) is extremely challenging and is rarely performed as part of routine care or drug surveillance. PZA requires an acidic environment (pH 5.5) for its activity, which affects the viability of mycobacteria and makes the test unreliable [4,9]. However, the BACTEC MGIT 960 system is considered the principal reference technique, involving an expensive instrument that limits its routine use in resource-poor settings, in addition to the false resistance results from growth inhibition by acidic liquid media [9]. The lack of PZA susceptibility testing in routine diagnosis impedes the effort to cure tuberculosis. For this reason, a reliable in-house assay for PZA susceptibility testing is essential for the identification and initiation of effective combination regimens.

Nicotinamide (NIC), a structural analogue of PZA with antituberculosis activity, is also converted to nicotinic acid by PZase at neutral pH [10,11]. Some colorimetric methods have utilized NIC as a surrogate for PZA to overcome the inhibitory effects of acidic culture media on bacterial growth. These include resazurin microtiter assay (REMA), nitrate reductase assay (NRA), microplate Alamar Blue assay (MABA), malachite green decolorization assay, and crystal violet decolorization assay. All assays returned reliable results with a good concordance between tuberculosis strains resistant to PZA and NIC [11,12,13,14,15]. The range of NIC concentrations varied between 8 to 2000 mg/L and 250 to 1000 mg/L in different assays with the lowest cut-off in REMA being >250 mg/L [12,13,14,15]. Several colorimetric redox dyes, as indicators of viability, have been used for rapid DST testing in *M. tuberculosis*, such as resazurin, 3-(4,5-dimethylthiazol-2-yl)-2,2,3,5-triphenyltetrazolium chloride (TTC), 5-cyano-2,3-ditolyl tetrazolium chloride (CTC), malachite green, and crystal violet [16,17,18]. Drug-resistant tuberculosis strains were easily observed by the color changes in the medium.

A biphasic medium was developed to increase the recovery of mycobacteria grown on solid or liquid media [19]. Moreover, the use of liquid media can enhance the growth rate of *M. tuberculosis*; a liquid culture has more inclination towards contamination compared to a solid culture and is less expensive than an automated liquid culture. Gonzalo et al. first developed a biphasic medium for PZA susceptibility testing, composed of Lowenstein–Jensen medium and a semisolid Kirchner medium, which has shown 95% reproducibility [20]. In this study, we aimed to develop a biphasic medium assay (BMA) to detect PZA resistance using NIC as a surrogate and evaluated the results with the BACTEC MGIT 960 method and *pncA* gene sequencing.

## 2. Results

### 2.1. Antimicrobial Susceptibility Testing

**Figure 1:**
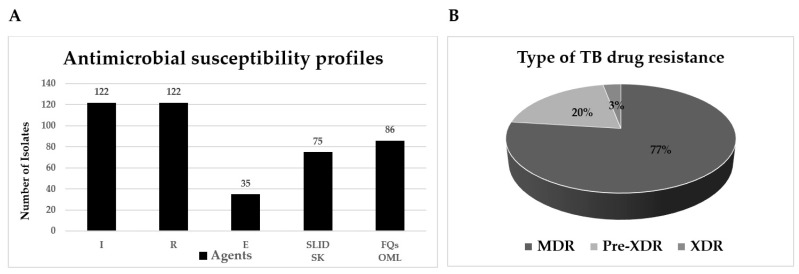
Antimicrobial susceptibility testing profiles by absolute concentration method of 122 MDR-TB isolates. (**A**) Percent of TB resistance in each drug or drug group. (**B**) Type of TB drug resistance (%). I, isoniazid; R, rifampicin; E, ethambutol; SLID, second line injectable drugs; S, streptomycin; K, kanamycin; FQs, fluoroquinolones; O, ofloxacin; M, moxifloxacin; L, levofloxacin; MDR, multidrug resistance; Pre-XDR, pre-extensive drug resistance; XDR, extensive drug resistance.

The antimicrobial susceptibility profiles of 121 MDR-TB are shown in Figure 1A. In the first line, oral anti-TB drugs revealed 100% IR resistance and 28.69% E resistance. Overall, 58.20% and 20.49% resisted the second-line injectable drugs (SLID) and fluoroquinolones (FQs), respectively. Pre-extensively drug-resistant TB (Pre-XDR-TB), defined as MDR-TB with resistance to fluoroquinolones (FLQs) or second-line injectable drugs (SLID), had 19% resistance. Extensively drug-resistant TB (XDR-TB), defined as MDR-TB with resistance to any fluoroquinolones and any of the SLID, had 3% (Figure 1B). 

### 2.2. Setting Up the Cut-Off Point of NIC-BMA for PZA Susceptibility Testing

The cut-off point of NIC concentration, to identify the PZA-resistant and -susceptible strains, were established by comparing results with the MGIT 960 reference method. As shown in Table 1, all 40 PZA-resistant isolates determined by MGIT 960, had minimum inhibitory concentrations (MICs) of NIC ≥ 1000 mg/L. Of all 121 PZA-susceptible isolates (82 MDR-TB and 39 drug-susceptible isolates) as determined by BACTEC MGIT 960, 4 (3.31%) showed discrepant NIC outcomes with MICs ≥ 1000 mg/L, while the remaining 117 (96.69%) of the drug-susceptible isolates had MICs ≤ 500 mg/L. Based on best-fit results between NIC-BMA and the MGIT 960, a cut-off value or “critical concentration” for MICs of NIC ≥ 1000 mg/L indicated PZA resistance. *M. tuberculosis* H37Rv and *M. bovis* BCG ATCC 37534 presenting PZA susceptibility (MIC of NIC ≤ 250 mg/L) and resistance (MIC of NIC > 2000 mg/L) as quality control strains, respectively. 

**Figure 2:**
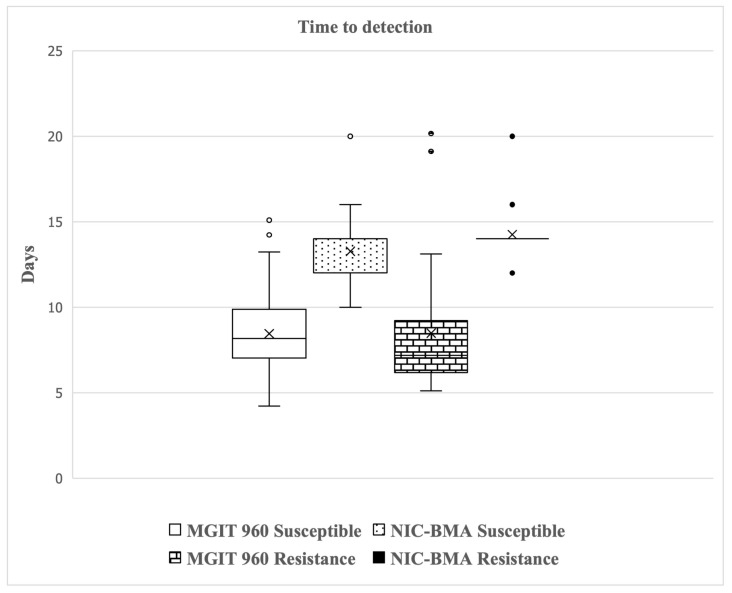
Detection time for pyrazinamide (PZA) susceptibility testing of 161 *Mycobacterium tuberculosis* isolates. A box plot shows the distribution of time to positive results by MGIT 960 system and nicotinamide biphasic medium assay (NIC-BMA) between PZA-susceptible and -resistant isolates. x, the median or the 50th percentile of each dataset; ○, outlier of datasets of MGIT 960; ●, outlier of datasets of NIC-BMA.

The detection time for PZA susceptibility testing of 161 MTB clinical isolates was compared between NIC-BMA and the MGIT 960 system, as shown in Figure 2. The average detection time with the MGIT 960 system and NIC-BMA were 9 days and 14 days, respectively. With the MGIT 960 system, the results were obtained after 4 to 20 days, while with NIC-BMA, results were obtained after 10 to 20 days. 

### 2.3. Validation of NIC-BMA and MGIT 960 System for PZA-Susceptibility Testing

As shown in Table 2, the comparison of NIC-BMA with MGIT 960 outcomes (40 resistant and 121 susceptible strains) yielded a sensitivity of 90.91% (95% confidence interval, 78.33% to 97.47%), a specificity of 100% (95% confidence interval, 96.90% to 100%), a positive predictive value of 100%, and a negative predictive value of 96.69% (95% confidence interval, 91.99% to 98.67%), with an accuracy of 97.52%.

### 2.4. pncA Gene Mutation

Only 122 MDR-TB isolates (40 PZA resistant and 82 PZA susceptible) were subjected to *pncA* gene sequencing. All MDR-TB isolates revealed 561 bp amplicon of *pncA* genes and its promoter; the electropherogram characteristics showed separated peaks of nucleotide sequences with an absence of background signals. We observed great mutant diversity in the *pncA* gene, as shown in Table 3. Thirty-seven of forty (92.5%) PZA-resistant isolates (defined by MGIT 960 and MIC of NIC ≥ 1000 mg/L) harbored a mutation within their *pncA* gene plus promoter region, including 21 isolates (52.5%) of amino acid substitutions, 5 isolates (12.5%) of its promoter mutations, 2 isolates (5%) of nonsense mutations, 1 isolate (2.5%) of double mutation, and 8 isolates (20%) of nucleotide insertion/deletion causing frameshift mutations. However, 3 of 40 (7.5%) of the PZA-resistant isolates had no *pncA* mutations. In this study, three novel mutations were identified from PZA-resistant strains (INS-97, INS-132, and DEL165-166). Seventy-eight of 82 (95.1%) PZA-susceptible isolates (defined by MGIT 960 and MIC of NIC ≤ 500 mg/L) were wild type (67, 81.7%) or susceptible associated with mutations (15, 18.3%). Three PZA-susceptible isolates with MIC of NIC ≥ 1000 mg/L had mutations (T61P, and F106L) with an unclear role in conferring PZA resistance and D136GR is mutation-associated resistant. Only one PZA-susceptible isolate showed discordance in *pncA* gene mutation-associated PZA resistance. 

## 3. Discussion

Pyrazinamide is a critical drug in both standard and novel regimens for tuberculosis treatment. However, the global prevalence of PZA resistance is increasing, especially for MDR-TB, with an estimated 270,000 new PZA-resistant cases annually, indicating that the implementation of PZA-susceptibility testing in routine tuberculosis laboratories is urgently needed [5].

Our new NIC-BMA was developed based on a colorimetric redox indicator assay using NIC as a surrogate for PZA DST and low-cost biphasic culture medium. The STC (2,3-diphenyl-5-thienyl-(2)-tetrazolium chloride) in NIC-BMA is a redox indicator that is a stable, water-insoluble dye that is not toxic to bacterial cells [21], in contrast to other tetrazolium compounds, including MTT 3-(4,5-dimethylthiazol-2-yl)-2,5-diphenyltetrazolium bromide, XTT 2,3-Bis-(2-methoxy-4-nitro-5-sulfophenyl)-2H-tetrazolium-5-carboxanilide and MTS (3-(4,5-dimethylthiazol-2-yl)-5-(3-carboxymethoxyphenyl)-2-(4-sulfophenyl)-2H-tetrazolium and CTC (5-cyano-2,3-ditolyl tetrazolium chloride) [22]. The benefits of colorimetric PZA DST over the proportional method are its convenience and reliability, enabling visual evaluation from obvious pink granule sediments with the naked eye [21]. Moreover, STC can be added simultaneously with the inoculum culture, which simplifies the process and thereby reduces the risk of infection during detection [16]. In some previous PZA DSTs, including REMA, NRA, MTT, and MABA, growth indicators had to be added to the culture after incubation, which increased the risk of mycobacterium transmission. The physiological activity of NIC at neutral pH overcomes the limitations of PZA and yields reliable results. The NIC cut-off concentration considered as reliable for the detection of resistance using NIC-BMA was established at MICs ≥ 1000 mg/L, whereas REMA needs > 250 mg/L [14], NRA [13], and MABA > 500 mg/L [12]. To establish the standard cut-off concentration, many samples from various geographic areas are required to cover the epidemiologic cut-off value of NIC.

The sensitivity and specificity of NIC-BMA were 90.91% and 100%, respectively, with no false-positive findings compared with the MGIT 960 reference method. Similarly, MABA using NIC and Middlebrook 7H9 broth yielded a sensitivity and specificity of 100% and 95.2%, respectively [12]. The NRA and REMA demonstrated sensitivities of 91% and 100% and specificities of 94% and 98%, respectively [13,14]. However, 4.88% (4/82) of PZA-susceptible isolates by MGIT 960 had MICs of NIC ≥ 1000 mg/L, with one *pncA* wild-type, two mutants with an unclear role in conferring PZA resistance (T61P and F106L) and one had mutation-associated resistance (D136G). False-PZA-susceptible outcomes from MGIT 960 were observed in many studies due to the growth rate of MTB being hindered in a lower pH medium [20]. In addition, in vitro PZase activities are influenced by starvation, energy inhibitors, reduced temperature, and microaerophilic or anaerobic conditions [3,23]. The reliability of NIC-BMA as MGIT 960 also depends on culture age, inoculum density, and inoculum homogeneity [13,24]. An inoculum with a lower cell density could prevent false resistance results and improve accuracy [9]. According to previous reports for *pncA* associated with DST outcomes [7,25,26,27], 37 of 40 PZA-resistant and 78 of 82 PZA-susceptible isolates had results of *pncA* mutations or wildtype consistent with previous findings. However, four PZA-susceptible isolates detected by MGIT 960, but with MICs of NIC ≥ 1000 mg/L, need further elucidation. 

A biphasic medium for PZA DST containing LJ solid medium and semisolid Kirchner medium with a critical concentration of 66 mg/L PZA at a pH of 5.2 to 5.5 had some major drawbacks, including a poor mycobacterial growth rate resulting in a long time to achieve results, of about 14 to 21 days [20]. The specificity of PZase testing increased for a prolonged incubation time of up to 10 days with the BacT/ALERT 3D system (range 2.57 to 20.29 days) [27]. For the MABA assay, the average time to results was after 9 days in comparison to BACTTEC MGIT 960 which were available after an average of 10 days [12]. For our NIC-BMA, the minimum time to results was 10 days, with an average of 13.52 days. The NIC-BMA formulation utilized Middlebrook 7H9 Broth supplemented with 10% oleic acid/albumin/dextrose/catalase (OADC) enrichment, promoting the growth of *M. tuberculosis* apart from the neutral pH culture [19]. In addition, STC could shorten the incubation time because crystal formazan is insoluble, enhancing color intensity and thereby making observation easier [16]. Contamination by nutrient-rich media was not found in our study. However, non-mycobacteria or fungi may be distinguished if a color change in the solid media from yellow-green to emerald green is observed and finally rot accompanying turbidity of the liquid medium appears [19]. 

Many diverse *pncA* mutations were detected in 37 of 40 (92.5%) PZA-resistant isolates and 15 of 82 (18.29%) PZA-susceptible isolates, and the majority were nonsynonymous. The crystal structure and kinetic study in the *pncA* mutants generated by site-directed mutagenesis suggested that Asp8, Lys96, and Cys138 motif were key residues for catalysis, and Asp49, His57, and His71 for Fe^2^+ binding site [28] Among 26 of the 34 mutation patterns associated with the PZA resistance phenotype, 14 were located in the promoter region and 12 in the cavity of the pyrazinamidase active sites [8,29,30,31,32]. The I31T mutants might not play a role in PZA resistance [22], found in nine and one PZA susceptible and resistant strains detected by MGIT-960, respectively. Three novel mutations were identified in this study, which required further characterization for their effects. The three PZA-resistant isolates with no *pncA* mutations might result from other resistance mechanisms such as mutations in other resistance-associated genes [3], insufficient drug uptake or efflux pump activity [31,33]. A systematic review reported global mutation frequencies and diversity of 641 mutations from 2760 PZA-resistant strains, including 79% missense mutations, 16% deletions and insertions, 3.4% mutations in promoters, and 18% wild type [7]. The percentages of *pncA* mutations in PZA-resistant and -susceptible isolates in our MDR-TB isolates were 92.5% and 18.29%, respectively, similar to global incidence rates [5,7]. Although mutations in *pncA* genes were strongly correlated with PZA resistance, no hot-spot regions or geographic variations were identified which would limit wide adoption [5,8]. About 35.14% (13/37) of PZA resistance in MDR-TB was pre-XDR-TB and XDR-TB, which encourages the establishment of rapid, cheap, and reliable PZA susceptibility testing in low-resource settings.

In this study, the NIC-BMA serves as a rapid and reliable method for PZA susceptibility testing, which could guide an appropriate treatment for multidrug resistant tuberculosis patients. However, our study was limited by the variations and numbers of PZA-resistant/-susceptible isolates which contributed to establishing the standard NIC cut-off concentration effectively. Multicenter studies with high numbers of isolates are required.

## 4. Materials and Methods

### 4.1. M. tuberculosis Isolates

A total of 161 *Mycobacterium tuberculosis* isolates were obtained from the Central Chest Institute of Thailand, Nonthaburi, Thailand. A sample size used in this study was calculated as the Buderer method which is used for the evaluation of the sensitivity and specificity of diagnostic tests at 95% confidence interval [34]. The routine drug susceptibility testing was performed by absolute concentration method with isoniazid (I, 0.2 and 1 µg/mL), rifampicin (R, 40 µg/mL), ethambutol (E, 2 µg/mL), streptomycin (S, 4 µg/mL), kanamycin (K, 30 µg/mL), ofloxacin (Ofx, 2 µg/mL), levofloxacin (Lfx, 2 µg/mL), moxifloxacin (Mfx, 0.75 µg/mL). All 122 MDR-TB isolates, which resisted at least isoniazid and rifampicin, and 39 drug-sensitive strains were collected consecutively from 2015 to 2018 and April 2022, respectively. The study was approved by the Ethical Committee of the Central Chest Institute of Thailand (certificate number 197/2561).

### 4.2. PZA Susceptibility Testing by BACTEC MGIT 960

PZA susceptibility testing was performed in a BACTEC MGIT 960 system with MGIT 960 PZA kits (BD Biosciences, Sparks, MD, USA) according to the manufacturer’s instructions. Bacterial isolates were prepared to the 0.5 McFarland standard. The bacterial suspension was diluted to 1:5 and 1:50 in sterile distilled water. The critical concentration of PZA used in this method was 100 mg/L. The 1:50 diluted suspension was inoculated into growth control tubes, and the 1:5 diluted suspension was inoculated at 0.5 mL into PZA tubes. For quality control, *M. tuberculosis* strain H37Rv and *M. bovis* ATCC 37534 were used as PZA-susceptible and PZA-resistant controls, respectively.

### 4.3. Biphasic Medium Assay (BMA)

**Figure 3:**
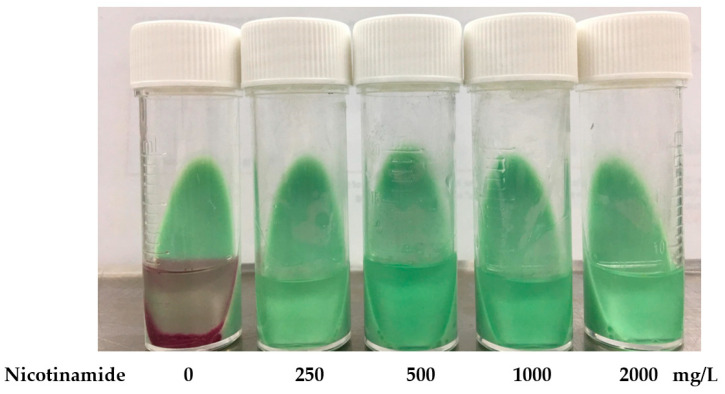
*Mycobacterium tuberculosis* H37Rv culture in a biphasic medium. Nicotinamide (NIC) was administered at concentrations of 250, 500, 1000, and 2000 mg/L. *M. tuberculosis* H37Rv was incubated in a biphasic medium at 37 °C for 21 days. Pink granule sediments indicate positive colonies of mycobacteria.

The biphasic medium consisted of 7 mL of LJ slant medium and 3 mL of Middlebrook 7H9 Broth with 10% OADC enrichment, prepared as described in Cui et al. [19]. NIC (Sigma-Aldrich, St. Louis, MO, USA) at final concentrations of 250, 500, 1000, and 2000 mg/L were solubilized in both solid and liquid phases of the medium. DST inoculum was prepared from 3- to 4-week-old bacterial cultures on LJ medium. Tuberculosis strains were suspended in 7 mL of sterile distilled water, homogenized by vigorous agitation in the presence of glass beads, and adjusted to a McFarland turbidity of 0.5. The 1:5 diluted suspension was inoculated into a biphasic medium containing NIC at each concentration and the growth control tube. Ten microliters of 3 mg/mL 2,3-diphenyl-5-thienyl-(2)-tetrazolium chloride (STC) (Tokyo Chemical Industry, Tokyo, Japan) was added to the liquid phase. All NIC-BMA and control tubes without NIC were incubated at 37 °C and observed daily for 21 days for visible colonies on LJ slant medium and/or pink granule sediments in the liquid medium (Figure 3). STC, a redox indicator that turns from colorless to pink granule sediments assembling around bacteria, indicated the growth of bacteria. The MIC for PZA-susceptible and PZA-resistant strains were determined. The lowest NIC concentration without sign of bacterial growth was determined as the MIC.

### 4.4. pncA Amplification and Sequencing

DNA was extracted from the MGIT culture in GC tubes of both PZA-susceptible and -resistant isolates by boiling. Briefly, 7 mL of MGIT culture was centrifuged at 3000× *g* for 15 min, suspended in 0.6 mL of TE buffer, and boiled for 20 min. DNA was precipitated by cold absolute ethanol. The primers used to amplify and sequence the 561 bp *pncA* gene and its promoter region were *pncA*-f (5′ GCACCAAGGCCGCGATGACAC 3′) and *pncA*-r (5′ CGCGCGTCACCG GTGAACAACC 3′). Polymerase chain reaction (PCR) amplification was performed as described previously [35]. PCR products were purified and sequenced using a BigDye terminator version 3.1 cycle sequence reaction kit and an ABI 3130 genetic analyzer (Applied Biosystems, Austin, TX, USA) as recommended in the manufacturer’s instructions. The obtained sequences were compared with the standard sequence from *M. tuberculosis* H37Rv using BioEdit version 7.2 software.

### 4.5. Statistical Analysis

The sensitivity, specificity, and accuracy of NIC-BMA in detecting PZA resistance in MDR-TB isolates were verified and evaluated in comparison with the BACTEC MGIT 960 method using the MedCalc online tool (https://www.medcalc.org/calc/diagnostic_test.php, accessed on 1 December 2019).

## 5. Conclusions

BMA using NIC serves as an inexpensive, and reliable DST for PZA susceptibility testing. This BMA can be easily prepared in laboratories using locally made media, which will be useful in limited-resource countries. Using STC as redox indicator minimized the risk of tuberculosis spreading during DST. The NIC-BMA for PZA susceptibility testing facilitates effective tuberculosis treatment and control.

## Figures and Tables

**Table 1:** Routine antimicrobial susceptibility testing, PZA susceptibility and MIC distribution of NIC-BMA in 161 *Mycobacterium tuberculosis* clinical isolates.

Routine DST Absolute Concentration (N)	PZA BACTEC MGIT 960 (N)	Number of Isolates at MIC of NIC-BMA				
		<250 mg/L	500 mg/L	1000 mg/L	2000 mg/L	>2000 mg/L
MDR-TB (122)	Resistance (40)	0	0	7	12	21
	Susceptible (82)	37	41	2	2	0
Susceptible (39)	Susceptible (39)	26	13	0	0	0

**Table 2:** Validation of PZA-susceptibility testing by NIC-BMA and BACTEC MGIT 960.

BACTEC MGIT 960	NIC-BMA MIC ≥ 1000 mg/L		
	PZA Resistance	PZA Susceptible	Total
PZA resistance	40	0	40
PZA susceptible	4	117	121
Total	44	117	161

Sensitivity: 90.91% CI 95% [78.33–97.47%]; Specificity: 100% CI 95% [96.90–100%]; Positive predictive value: 100%; Negative predictive value: 96.69% CI 95% [91.99–98.67%]; Accuracy: 97.52% CI 95% [93.76–99.32%].

**Table 3:** PZA susceptibility results from the MGIT 960 method, NIC-BMA MIC distribution, and *pncA* and promoter-region sequencing in 122 MDR-TB isolates.

MGIT 960 (No. of Isolates)	*pncA* Characterization (No. of Isolates)	*pncA* and Promoter Region Sequencing Outcomes (No. of Isolates)	NIC-BMA MIC (mg/L) (No. of Isolates)
Resistant (40)	Mutant *pncA* (37) Promoter (5) Nonsynonymous (21) Insertion/deletion (8) Double mutation (1) Nonsense (2) Wild-type *pncA* (3)	-	<250 (0)
		-	500 (0)
		A-11C ^R^ (1), D12A ^R^ (1), Y41STOP ^R^ (1), P62L ^R^ (1), INS-97 ^N^ (1), Y103STOP ^R^ (1), INS-150 ^R^ (1)	1000 (7)
		A-11G ^R^ (1), D12A ^R^ (1), T47P ^R^ (1), I90S ^R^ (1), G105V ^R^ (1), INS-132 ^N^ (1), INS-136 ^R^ (2), V139L ^R^ (1), INS-177 ^R^ (1), wild-type(2)	2000 (12)
		A-11C ^R^ (2), A-11G ^R^ (1), G24D ^R^ (1), L35P ^R^ (1), S67P ^R^ (1), W68G ^R^ (1), C72Y ^R^ (1), I90S ^R^ (2), S104R ^R^ (2), W119R ^R^ (1), L120R ^R^ (1), D126H + D129Y ^R^ (1), INS-136 ^R^ (1), V139A ^R^ (1), V139G ^R^ (1), DEL165-166 ^N^ (1), L172P ^R^ (1), wild-type(1)	>2000 (21)
Susceptible (82)	Mutant *pncA* (15) Promoter (0) Nonsynonymous (15) Insertion/deletion (0) Wild-type *pncA* (67)	I31T ^S^ (4), D63P ^N^ (1), wild-type (32)	<250 (37)
		I31T ^S^ (3), T61P ^I^ (3), V163A ^S^ (1), wild-type (34)	500 (41)
		D136G ^R^ (1), wild-type (1)	1000 (2)
		T61P ^I^ (1), F106L ^I^ (1)	2000 (2)
		-	>2000 (0)

^S^ Cautiously susceptible, associated with mutations. ^R^ Resistant, associated with mutations. ^I^ Mutations with an unclear role in conferring PZA resistance. ^N^ No data available; MIC, minimum inhibitory concentration; NIC-BMA, nicotinamide biphasic medium assay; PZA, pyrazinamide; INS, insertion; DEL, deletion.

## Data Availability

The data presented in this study are available on request.
